# Pregnancy in Autosomal Dominant Polycystic Kidney Disease: Maternal and Fetal Considerations: A Narrative Review

**DOI:** 10.1155/jp/9166037

**Published:** 2026-07-19

**Authors:** Pooneh Jabbaripour Sarmadian, Amir Dolatshahi Pirooz, Maha Al Baghdadi, Mohammad Kamgar, Yalda Afshar, Niloofar Nobakht

**Affiliations:** ^1^ Division of Endocrinology, Diabetes and Metabolism, Johns Hopkins University School of Medicine, Baltimore, Maryland, USA, jhu.edu; ^2^ Beverly Hills Cancer Center, Clinical Research, Los Angeles, California, USA; ^3^ Division of Nephrology, Department of Medicine, Kaiser Permanente Downey Medical Center, Downey, California, USA; ^4^ Division of Nephrology, Department of Medicine, David Geffen School of Medicine, University of California Los Angeles, Los Angeles, California, USA, ucla.edu; ^5^ Division of Maternal Fetal Medicine, Department of Obstetrics and Gynecology, David Geffen School of Medicine, University of California Los Angeles, Los Angeles, California, USA, ucla.edu

**Keywords:** ADPKD, hypertension, maternal outcomes, preeclampsia, pregnancy

## Abstract

**Background and Aims:**

Autosomal dominant polycystic kidney disease (ADPKD) is the most common inherited kidney disorder and an important cause of kidney failure among individuals of reproductive age. Although its management in nonpregnant adults is well established, the implications of ADPKD during pregnancy remain less clearly defined. The aim of this review is to summarize maternal and fetal risks and provide practical management considerations for pregnancy in individuals with ADPKD.

**Methods:**

A narrative review of the literature was conducted using PubMed/MEDLINE and relevant clinical practice guidelines, focusing on pregnancy‐related outcomes and management considerations in individuals with ADPKD.

**Results:**

Pregnant individuals with ADPKD face higher risks of chronic hypertension, proteinuria, hypertensive disorders of pregnancy, and preterm delivery compared with the general population. Strict blood pressure targets used outside of pregnancy may compromise uteroplacental perfusion, highlighting the importance of pregnancy‐appropriate goals and safe antihypertensive choices. Renin–angiotensin system inhibitors and disease‐modifying therapies such as tolvaptan are contraindicated due to fetal safety concerns. Monitoring strategies include regular assessment of blood pressure, kidney function, and fetal growth. Emerging imaging data suggest that pregnancy may accelerate kidney and liver cyst growth, although long‐term consequences remain uncertain. Additional strategies such as low‐dose aspirin, biochemical markers for preeclampsia risk assessment, and renal ultrasound for baseline evaluation may be considered.

**Conclusion:**

Pregnancy in individuals with ADPKD requires individualized, multidisciplinary care with careful attention to blood pressure control, medication safety, and maternal and fetal monitoring. Further research is needed to better understand long‐term outcomes and optimize management strategies.

**Key Points**

• Pregnant individuals with ADPKD have a higher risk of chronic hypertension, proteinuria, hypertensive diseases of pregnancy, and preterm delivery compared with the general obstetric population.
• In nonpregnant individuals with ADPKD, intensive blood pressure control such as < 110/75 mm Hg may be recommended. In pregnant individuals with pre‐existing hypertension, a target blood pressure of < 140/90 mm Hg is generally recommended to improve pregnancy outcomes.
• Coordinated care with regular monitoring of maternal health, fetal growth, and discussions about inheritance and genetic testing can help support the best outcomes for both parent and baby.

## 1. Introduction

**Figure 1:**
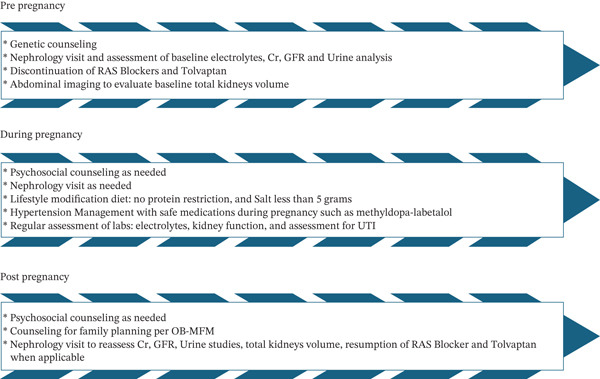
Suggested management approach for women with autosomal dominant polycystic kidney disease (ADPKD) during the prepregnancy, pregnancy, and postpartum periods.

Autosomal dominant polycystic kidney disease (ADPKD) is the most common inherited kidney disorder, affecting approximately 1 in every 1000 individuals worldwide [1, 2]. It is characterized by progressive cyst formation, kidney enlargement, and eventual loss of kidney function, with a significant proportion of patients developing kidney failure later in life [2]. Although individuals with ADPKD may remain asymptomatic through their reproductive years, pregnancy can unmask or worsen underlying manifestations of the disease [3, 4]. Evidence suggests that pregnant individuals with ADPKD have higher risks of hypertension, proteinuria, preeclampsia, and preterm delivery compared with the general obstetric population [3, 5]. This review summarizes current knowledge on pregnancy in ADPKD, focusing on maternal and fetal risks, hypertension management, medication considerations, imaging surveillance, cyst growth, and genetic implications. Key clinical considerations are summarized in Table 1, and a suggested management approach for women with ADPKD during the prepregnancy, pregnancy, and postpartum periods is presented in Figure 1.

**Table 1:** Summary of key clinical considerations for pregnancy in ADPKD.

Domain	Key considerations
Prepregnancy counseling	Discuss the autosomal dominant inheritance pattern and the 50% transmission risk; offer genetic counseling and discuss prenatal or preimplantation genetic testing options; review current medications and discontinue teratogenic agents prior to conception.
Blood pressure management	Use pregnancy‐appropriate blood pressure targets; intensive targets used outside pregnancy, such as < 110/75 mm Hg, are not recommended. Blood pressure goals should be individualized during pregnancy. Current SMFM recommendations generally support treatment to maintain blood pressure below 140/90 mm Hg, whereas CKD‐specific pregnancy guidelines recommend targets below 135/85 mm Hg. Preferred antihypertensive agents include labetalol, nifedipine, and methyldopa; renin–angiotensin system inhibitors must be discontinued because of fetal risk.
Medication safety	Tolvaptan is contraindicated during pregnancy and breastfeeding; renin–angiotensin system blockers should be avoided; antihypertensive therapy should be selected based on established pregnancy safety profiles.
Maternal monitoring	Regular monitoring of blood pressure, serum creatinine, and proteinuria is recommended; monitoring frequency should be individualized based on baseline kidney function, cyst burden, and the presence of hypertension.
Acute complications	Cyst infection, cyst hemorrhage, and nephrolithiasis should be considered in pregnant individuals presenting with flank pain, fever, or hematuria; management should be individualized, and pregnancy‐safe imaging modalities should be used when indicated.
Imaging considerations	Renal ultrasound is recommended if not performed within the prior year to establish a baseline and assess cyst burden; additional imaging should be guided by clinical indications.
Intracranial aneurysms	Consider preconception magnetic resonance angiography screening in high‐risk individuals; available evidence suggests that the presence of an intracranial aneurysm alone does not generally mandate a specific mode of delivery.
Fetal surveillance	Serial fetal ultrasound assessments are recommended to monitor fetal growth, amniotic fluid volume, and placental function, particularly in the third trimester.
Preeclampsia risk reduction	Low‐dose aspirin is recommended for individuals at increased risk; biomarker‐based assessment using the sFlt‐1/PlGF ratio may assist in preeclampsia risk stratification when clinically indicated.
Cyst growth considerations	Pregnancy may be associated with accelerated kidney and liver cyst growth; the long‐term clinical significance of these changes remains uncertain and warrants continued follow‐up.
Delivery planning	Timing and mode of delivery should be individualized based on maternal disease severity, fetal status, and the development of complications such as severe preeclampsia.
Postpartum care	Reassess blood pressure and kidney function postpartum; disease‐modifying therapy and renin–angiotensin system inhibitors may be resumed after breastfeeding if clinically indicated; provide contraception counseling, favoring progesterone‐only or nonhormonal methods.
Multidisciplinary care	Close coordination between nephrology and maternal–fetal medicine teams is essential throughout pregnancy, delivery, and postpartum care.

## 2. Methods

This narrative review summarizes the current literature on pregnancy in individuals with ADPKD. Relevant studies were identified through searches of PubMed/MEDLINE from January 1994 through May 2026 using combinations of the terms “autosomal dominant polycystic kidney disease,” “ADPKD,” “pregnancy,” “maternal outcomes,” “fetal outcomes,” “hypertension,” “preeclampsia,” “tolvaptan,” and “intracranial aneurysm.” Additional sources were identified through review of relevant clinical practice guidelines, including the KDIGO 2025 ADPKD guideline [6], ACOG Practice Bulletin No. 203 on chronic hypertension in pregnancy [7], and the Society for Maternal–Fetal Medicine statement on antihypertensive therapy in pregnancy [8], as well as reference lists of key publications.

English‐language articles addressing maternal and fetal outcomes, blood pressure management, medication safety, intracranial aneurysm screening, and monitoring during pregnancy in individuals with ADPKD were reviewed. Eligible publications included case reports, observational studies, clinical trials, review articles, and guideline statements that were relevant to the clinical management of pregnancy in ADPKD. Studies that did not specifically address ADPKD or pregnancy, as well as publications without accessible full‐text versions, were excluded. Because large prospective studies in this area remain limited, both landmark and more recent publications were included, with greater weight given to the most up‐to‐date evidence when differences in findings existed.

### 2.1. Hypertension and Preeclampsia

Hypertension is one of the earliest clinical manifestations of ADPKD and may appear long before overt kidney dysfunction [1, 2, 9].

Pregnancy further increases the risk of hypertensive disorders, with studies showing up to a twofold increase in preeclampsia among individuals with ADPKD compared with unaffected pregnancies [5, 10]. Strict blood pressure targets commonly used outside pregnancy may compromise uteroplacental perfusion and are not appropriate during pregnancy [7]. For pregnant individuals with chronic kidney disease, most guidelines recommend maintaining blood pressure below 135/85 mmHg [4], whereas the Society for Maternal–Fetal Medicine recommends a treatment goal of < 140/90 mmHg in pregnant individuals with chronic hypertension [8]. These differing thresholds reflect variations in guideline recommendations and patient populations, and blood pressure goals should be individualized according to maternal renal function and obstetric risk factors. In contrast, renin–angiotensin system inhibitors must be discontinued because of the risk of congenital anomalies and fetal kidney injury [4].

### 2.2. Intracranial Aneurysms (IAs) and Pregnancy Considerations

IAs are more prevalent in individuals with ADPKD, with an estimated prevalence of approximately 9%–12% compared with 2%–3% in the general population [11, 12]. Although most aneurysms remain asymptomatic, rupture can lead to subarachnoid hemorrhage, a rare but potentially life‐threatening complication associated with significant maternal morbidity and mortality during pregnancy [11, 13].

Screening is typically performed using noninvasive imaging such as magnetic resonance angiography and is ideally done during preconception counseling, prior to pregnancy in high‐risk individuals [12]. In the context of pregnancy, current evidence does not clearly demonstrate a substantial increase in the risk of aneurysm rupture, although physiological hemodynamic and hormonal changes during pregnancy may theoretically affect vascular dynamics and aneurysm behavior [12, 13].

The presence of an IA alone does not generally mandate a specific mode of delivery, and delivery should generally be determined based on obstetric indications [12, 13].

A multidisciplinary approach involving neurology, neurosurgery, and maternal fetal medicine is recommended to guide individualized risk assessment and delivery planning [8].

### 2.3. Medication Considerations

Tolvaptan, the only FDA‐approved disease‐modifying therapy for slowing ADPKD progression, is not recommended during pregnancy or breastfeeding because of limited human safety data and concerning findings from preclinical studies [14, 15].

There are no human safety data supporting the use of angiotensin receptor antagonists in pregnancy, so discontinuation prior to conception is recommended [3]. Individuals who require renin–angiotensin system blockade for strong indications should be counseled to stop these medications immediately once pregnancy is confirmed [4].

### 2.4. Monitoring and Imaging

Monitoring during pregnancy should be individualized based on baseline kidney function, cyst burden, and the presence of hypertension [3]. Frequent assessment of blood pressure, serum creatinine, and proteinuria is recommended, particularly for individuals with pre‐existing kidney impairment [3, 4]. A renal ultrasound is advisable if one has not been performed within the past year, as it provides a baseline for monitoring cyst size and identifying complications such as cyst rupture or infection [3]. Fetal ultrasound remains a key tool for assessing fetal anomalies and uteroplacental insufficiency, including fetal growth restriction and oligohydramnios [3, 4].

### 2.5. Acute Complications in Pregnancy

Individuals with ADPKD may experience acute complications during pregnancy, including cyst infection, cyst hemorrhage, and urolithiasis [3, 4].

These complications may present with abdominal or flank pain, fever, or hematuria and may mimic other pregnancy‐related renal or abdominal conditions [3, 16]. Cyst infection should be suspected in patients with persistent fever and localized pain, and management typically includes appropriate antibiotic therapy with agents that are safe in pregnancy [3].

Imaging with renal ultrasound is usually preferred as an initial modality, whereas magnetic resonance imaging may be considered when the diagnosis remains uncertain [3].

Cyst hemorrhage may present with acute pain and sometimes gross hematuria and is often managed conservatively with pain control and supportive care [3].

Urolithiasis may also occur and should be considered in the differential diagnosis of flank pain, particularly in pregnant individuals with ADPKD [16].

Ultrasound is the first‐line imaging modality, and management depends on the severity of symptoms and presence of obstruction or infection [4].

Early recognition and appropriate management of these complications are important to reduce maternal morbidity and avoid adverse pregnancy outcomes [3].

### 2.6. Pregnancy‐Associated Cyst Growth

Pregnancy may influence the progression of renal manifestations in individuals with ADPKD because of physiological and hemodynamic changes associated with pregnancy [16]. Limited review‐level evidence suggests that pregnancy may be associated with increased kidney and liver cyst growth in individuals with ADPKD [17]. Although the physiological and hormonal changes associated with pregnancy may contribute to disease manifestations, the available evidence does not clearly demonstrate that pregnancy accelerates long‐term kidney function decline [18]. More research is needed to determine whether these changes contribute to long‐term disease progression.

### 2.7. Genetic Considerations

ADPKD is primarily caused by pathogenic mutations in PKD1 or PKD2, with PKD1 mutations typically associated with more severe disease and earlier onset of kidney failure [2, 17, 18].

Individuals with more advanced diseases at conception may face higher risks of complications during pregnancy [19]. Mutation location within PKD2 may also influence clinical outcomes, although data are limited [20]. Genetic counseling is an important component of prepregnancy preparation because ADPKD is inherited in an autosomal dominant pattern, giving each child a 50% chance of inheriting the disease [1, 19]. Prenatal diagnostic options and preimplantation genetic testing may be considered for those who wish to assess transmission risk [21].

### 2.8. Fetal Considerations

Despite increased maternal risks, most pregnancies in individuals with ADPKD result in healthy newborns [3, 5]. In pregnancies complicated by chronic kidney disease, maternal hypertension and impaired kidney function are associated with increased risks of fetal growth restriction and preterm delivery [19]. Ultrasound surveillance is recommended throughout pregnancy [3, 4]. Although ADPKD is inherited in an autosomal dominant pattern, manifestations are not typically present until adulthood, and neonatal complications are uncommon outside rare cases of early severe disease [1, 2].

### 2.9. Guidelines and Clinical Recommendations

The 2025 KDIGO guidelines emphasize individualized monitoring, including early initiation of low‐dose aspirin from 12 to 36 weeks to reduce the risk of preeclampsia [6]. Clinical guidelines note that the soluble fms‐like tyrosine kinase‐1‐to‐placental growth factor ratio may be used beginning at 24 weeks and repeated every 4–6 weeks to support preeclampsia risk assessment, although its use is not routine in all clinical settings [6]. Coordination between nephrology and maternal–fetal medicine teams is essential for managing medication transitions, monitoring kidney function, and planning delivery timing [22].

### 2.10. Delivery and Postpartum Care

Delivery timing depends on maternal disease severity, fetal status, and the presence of complications such as hypertensive disorders of pregnancy [3].

In general, the mode of delivery is dictated by routine obstetrical indications rather than the presence of ADPKD [3]. Postpartum care includes reassessment of blood pressure, renal function, and medication needs [3, 4]. Renin–angiotensin system inhibitors and tolvaptan may be resumed after breastfeeding has ended or if the individual is not breastfeeding [3, 4, 14]. Contraception counseling is important, with progesterone‐only or nonhormonal methods preferred in those with chronic kidney disease [4].

## 3. Conclusion

Pregnancy in individuals with ADPKD carries increased maternal and fetal risks and requires careful, multidisciplinary management, including blood pressure control, avoidance of teratogenic medications, tailored monitoring strategies, and genetic counseling. Although recent studies provide insights into pregnancy‐related cyst growth and genotype–phenotype relationships, further research is needed to clarify long‐term maternal and fetal outcomes.

## Funding

No funding was received for this manuscript.

## Disclosure

All authors have read and approved the final version of the manuscript. The corresponding author takes full responsibility for the integrity of the work. All authors affirm that this manuscript is an accurate and transparent account of the work being reported and that no important aspects have been omitted.

## Ethics Statement

This review did not involve human subjects, animals, or patient data and did not require institutional review board approval.

## Conflicts of Interest

The authors declare no conflicts of interest.

## Data Availability

Data sharing is not applicable to this article as no new data were created or analyzed.
